# The effect of adenosine deaminase inhibition on the A_1_ adenosinergic and M_2_ muscarinergic control of contractility in eu- and hyperthyroid guinea pig atria

**DOI:** 10.1007/s00210-015-1121-6

**Published:** 2015-04-16

**Authors:** Krisztian Pak, Judit Zsuga, Zita Kepes, Tamas Erdei, Balazs Varga, Bela Juhasz, Andras Jozsef Szentmiklosi, Rudolf Gesztelyi

**Affiliations:** Department of Pharmacology, Faculty of Pharmacy, University of Debrecen, Nagyerdei krt. 98, 4032 Debrecen, Hungary; Department of Health Systems Management and Quality Management for Health Care, Faculty of Public Health, University of Debrecen, Nagyerdei krt. 98, 4032 Debrecen, Hungary; Department of Pharmacology and Pharmacotherapy, Faculty of Medicine, University of Debrecen, Nagyerdei krt. 98, 4032 Debrecen, Hungary

**Keywords:** Adenosine deaminase inhibition, Thyroid hormones, Heart, Atrium, Guinea pig, RRM

## Abstract

The A_1_ adenosine and M_2_ muscarinic receptors exert protective (including energy consumption limiting) effects in the heart. We investigated the influence of adenosine deaminase (ADA) inhibition on a representative energy consumption limiting function, the direct negative inotropic effect elicited by the A_1_ adenosinergic and M_2_ muscarinergic systems, in eu- and hyperthyroid atria. Furthermore, we compared the change in the interstitial adenosine level caused by ADA inhibition and nucleoside transport blockade, two well-established processes to stimulate the cell surface A_1_ adenosine receptors, in both thyroid states. A classical isolated organ technique was applied supplemented with the receptorial responsiveness method (RRM), a concentration estimating procedure. Via measuring the contractile force, the direct negative inotropic capacity of N^6^-cyclopentyladenosine, a selective A_1_ receptor agonist, and methacholine, a muscarinic receptor agonist, was determined on the left atria isolated from 8-day solvent- and thyroxine-treated guinea pigs in the presence and absence of 2′-deoxycoformycin, a selective ADA inhibitor, and NBTI, a selective nucleoside transporter inhibitor. We found that ADA inhibition (but not nucleoside transport blockade) increased the signal amplification of the A_1_ adenosinergic (but not M_2_ muscarinergic) system. This action of ADA inhibition developed in both thyroid states, but it was greater in hyperthyroidism. Nevertheless, ADA inhibition produced a smaller rise in the interstitial adenosine concentration than nucleoside transport blockade did in both thyroid states. Our results indicate that ADA inhibition, besides increasing the interstitial adenosine level, intensifies the atrial A_1_ adenosinergic function in another (thyroid hormone-sensitive) way, suggesting a new mechanism of action of ADA inhibition.

## Introduction

With the exception of the low-income countries, diseases affecting the cardiovascular system, primarily ischemic heart disease and stroke, represent the biggest challenge in terms of life expectancy (WHO 2014). Therefore, a basic understanding of endogenous protective mechanisms of the heart against ischemia is a prerequisite for the development of new rational therapeutic strategies (Perricone and Vander Heide 2014; Kleinbongard and Heusch 2014). One powerful endogenous protective mechanism in the living tissues is adenosinergic signaling, a part of the ancient and ubiquitous purinergic system (Verkhratsky and Burnstock 2014).

Effects of adenosine, a nucleoside of nucleic acid metabolism, are mediated predominantly by activating G protein-coupled adenosine receptors (Fredholm et al. 2001, 2011). The main adenosine receptor type in the heart is the A_1_ adenosine receptor (A_1_ receptor), which is involved in extensive protective and regenerative functions including negative tropic effects that limit energy consumption and thereby contribute to the protection of the heart against ischemic–hypoxic damages (Headrick et al. 2011, 2013). Pharmacological activation of the A_1_ adenosinergic system is a prospective, although yet modestly utilized, possibility that may have preventive and therapeutic implications in numerous cardiovascular maladies including ischemic heart disease and certain types of arrhythmias (Szentmiklosi et al. 2011, 2015); furthermore, it may come into play in avoiding hypoxic injury during heart transplantation (Lim et al. 2013; Burnstock and Pelleg 2014).

The thyroid state influences several regulatory mechanisms including functions of the A_1_ receptor (for a brief review, see Gesztelyi et al. 2012). Among others, thyroid hormones (T_3_, T_4_) markedly reduce the direct negative inotropic effect (decrease of the contractile force without prior positive inotropic stimulation exerted by another agent) of A_1_ receptor agonists (Szentmiklosi et al. 1992; Kaasik et al. 1994; Gesztelyi et al. 2003a). Hyperthyroidism is a pathological condition that, by upregulating a wide range of metabolic processes, raises the oxygen and nutrient consumption in the tissues and thus increases the work of the heart (Cini et al. 2009; Nabbout and Robbins 2010). As a consequence, excess thyroid hormones increase the risk of congestive heart failure, ischemic heart disease, and arrhythmias and thereby elevate cardiovascular mortality (Franklyn and Boelaert 2012). In light of these facts, suppression of the A_1_ adenosinergic system by thyroid hormones may raise concerns. Thus, it is of importance to find out possibilities to enhance the depressed function of the A_1_ adenosinergic system in hyperthyroidism.

An opportunity to influence the interstitial adenosine concentration is to blunt enzymes participating in the elimination of adenosine. Adenosine deaminase (ADA), by converting adenosine into inosine, plays an important, although not pivotal, role in the nucleic acid metabolism of the heart (Fredholm et al. 2001, 2011; Headrick et al. 2011). Inhibition (or deficiency) of ADA increases both the intra- and extra-cellular adenosine concentrations that enables the surplus adenosine to exert its effects via both intra- and extra-cellular binding sites (Sandhu et al. 1993; Zhu et al. 1994; Hudspeth et al. 1994; Manthei et al. 1998; Peart et al. 2001, 2003; Willems et al. 2006; Abd-Elfattah et al. 2012). Consistently, the inhibition of ADA augments actions of exogenous adenosine as well (Szentmiklosi et al. 1982; Gesztelyi et al. 2003b).

In a previous study, we found that inhibition of ADA increases the signal amplification of the A_1_ adenosinergic system regarding its direct negative inotropic function in the hyperthyroid guinea pig atrium (Kemeny-Beke et al. 2007). As ADA inhibition elevates the adenosine levels and thus augments all A_1_ receptor-mediated processes, it is not easy to identify this particular (efficiency-enhancing) action of ADA inhibition. Thus, the major goal of the present study was to develop an experimental setup suitable to gain further insight into the functional consequences of ADA inhibition as regards the A_1_ receptor-mediated direct negative inotropy under both eu- and hyperthyroid conditions.

Another way to manipulate the interstitial adenosine level is inhibition of the adenosine flux across the cell membrane. In the metabolically intact myocardium, adenosine mainly forms in the interstitium and is eliminated in the cell interior; therefore, net adenosine transport is directed into the cardiomyocytes (Deussen et al. 1999; Deussen 2000a, 2000b). In the heart, the transmembrane adenosine flux passes almost exclusively through ENT1, a nucleoside transporter type that is equilibrative and sensitive to inhibition by NBTI (Thorn and Jarvis 1996). Accordingly, in our previous studies, NBTI was found to elevate the interstitial adenosine level in the guinea pig atrium (Karsai et al. 2006; Kiss et al. 2013), an action which was more pronounced in hyperthyroidism (Karsai et al. 2007; Pak et al. 2014).

As the ligand binding site of adenosine receptors is accessible from the interstitium, it is especially important to gather information about changes of the interstitial adenosine concentration. However, in the functioning heart, these data cannot be assessed with sufficient accuracy (at least for our purposes) by the commonly used methods because of the rapid turnover and poor access of adenosine in the living tissues (Karsai et al. 2006; Ramakers et al. 2008). Under well-defined circumstances, the receptorial responsiveness method (RRM), a method that has been recently developed but is rooted in the classical pharmacology (Gesztelyi et al. 2004), may address this problem. RRM is based on a simplified mathematical modelling of the interaction between two agonists that consume the response capacity of the same (or at least greatly overlapping) signal transduction (Grenczer et al. 2010a, 2010b). This way, RRM enables the quantification of an acute increase in the concentration of an agonist via generating concentration–effect (*E*/*c*) curves with the same or another agonist (the latter of which is more stable or preferred for any other reasons) in the given tissue. As a limitation, if the two agonists are different, the surplus concentration in question can be quantified only with a surrogate parameter, i.e., the equieffective concentration of the other agonist. However, a unique feature of RRM is that, owing to its functional assay nature, it provides information about the agonist concentration in the vicinity of the specific receptors, a tissue compartment otherwise difficult to access in a working organ (Gesztelyi et al. 2004; Grenczer et al. 2010a, 2010b). Although RRM, in principle, can be applied for each receptor, the A_1_ receptor is especially suitable for this purpose due to its slow desensitization (Mundell and Kelly 2011) relative to the duration of the measurement. Thus, another goal of the present study was to assess the alteration in the interstitial adenosine level caused by ADA inhibition by means of RRM and then to compare it with the change produced by nucleoside transport blockade under both eu- and hyperthyroid conditions.

## Methods

### Materials

The following chemicals were used: l-thyroxine sodium salt pentahydrate (T_4_), adenosine (non-selective adenosine receptor full agonist), acetyl-β-methylcholine chloride (methacholine: MC; non-selective muscarinic receptor full agonist with high affinity for the M_2_ muscarinic receptor (M_2_ receptor)), N^6^-cyclopentyladenosine (CPA; selective A_1_ adenosine receptor full agonist), 8-cyclopentyl-1,3-dipropylxanthine (CPX; selective, competitive A_1_ adenosine receptor antagonist), and *S*-(2-hydroxy-5-nitrobenzyl)-6-thioinosine (NBTI; selective inhibitor of nucleoside transporter type ENT1) from Sigma (St. Louis, MO, USA) and pentostatin (2′-deoxycoformycin: DCF; selective inhibitor of adenosine deaminase) in Nipent^™^, which was the generous gift of Wyeth Pharmaceuticals (Collegeville, PA, USA).

Experiments were conducted in modified Krebs–Henseleit buffer (Krebs solution) that contained 118 mM NaCl, 4.7 mM KCl, 2.5 mM CaCl_2_, 1 mM NaH_2_PO_4_, 1.2 mM MgCl_2_, 24.9 mM NaHCO_3_, 11.5 mM glucose and 0.1 mM ascorbic acid (dissolved in redistilled water). T_4_ was dissolved in physiological salt solution containing 0.01 % NaOH. Adenosine and MC were dissolved at 36 °C in Krebs solution. CPA was dissolved in ethanol/water (1:4) solution (*v*/*v*). CPX and NBTI were dissolved in dimethyl-sulfoxide (DMSO). DCF was dissolved in redistilled water (according to the manufacturer’s instructions). Stock solutions were diluted with Krebs solution (when appropriate). The concentration of ethanol and DMSO did not exceed 0.023 % (*v*/*v*) and 0.1 % (*v*/*v*), respectively, in the organ baths at any time.

### Animals and preparations

All animal use protocols were approved by the Committee of Animal Research, University of Debrecen, Hungary (3/2012/DE MÁB). Male Hartley guinea pigs weighting 600–800 g were used. A group of animals received 330 μg/kg T_4_ daily (ip.) for 8 days (in vivo T_4_ treatment), while the vehicle of T_4_ was administered daily (ip.) for 8 days to the other group (in vivo solvent treatment). On the ninth day, the animals were guillotined; left atria were quickly removed and mounted at 10 mN resting tension in 10-ml vertical organ chambers (Experimetria TSZ-04) containing Krebs solution gassed with 95 % O_2_ and 5 % CO_2_ (36 °C; pH 7.4). Atria were paced by platinum electrodes (3 Hz, 1 ms, twice the threshold voltage) by means of a programmable stimulator (Experimetria ST-02) and power amplifier (Experimetria PST-02). The contractile force was characterized by the amplitude of the isometric twitches, which were detected by a transducer (Experimetria SD-01) and strain gauge (Experimetria SG-01D), and recorded by a polygraph (Medicor R-61 6CH Recorder).

### Experimental groups and protocols

First, all atria were allowed to equilibrate in Krebs solution for 40 min. Then, a cumulative concentration–effect (*E*/*c*) curve was constructed with adenosine. After a washout period (Krebs solution for 15 min), atria were randomized into groups for the subsequent in vitro treatment. In the group names, the applied in vivo and in vitro treatments (S—solvent-treated, T—T_4_-treated, Co—control) and abbreviations of the chemicals used were indicated: S Co (*n* = 16), T Co (*n* = 19), S CPX (*n* = 12), T CPX (*n* = 9), S DMSO (*n* = 6), T DMSO (*n* = 7), S NBTI (*n* = 6), T NBTI (*n* = 7), S DCF (*n* = 10), T DCF (*n* = 18), S DCF CPX (*n* = 8), T DCF CPX (*n* = 9), S Co (CPA) (*n* = 7), T Co (CPA) (*n* = 9), S DCF (CPA) (*n* = 7), and T DCF (CPA) (*n* = 10).

**Fig. 1:**
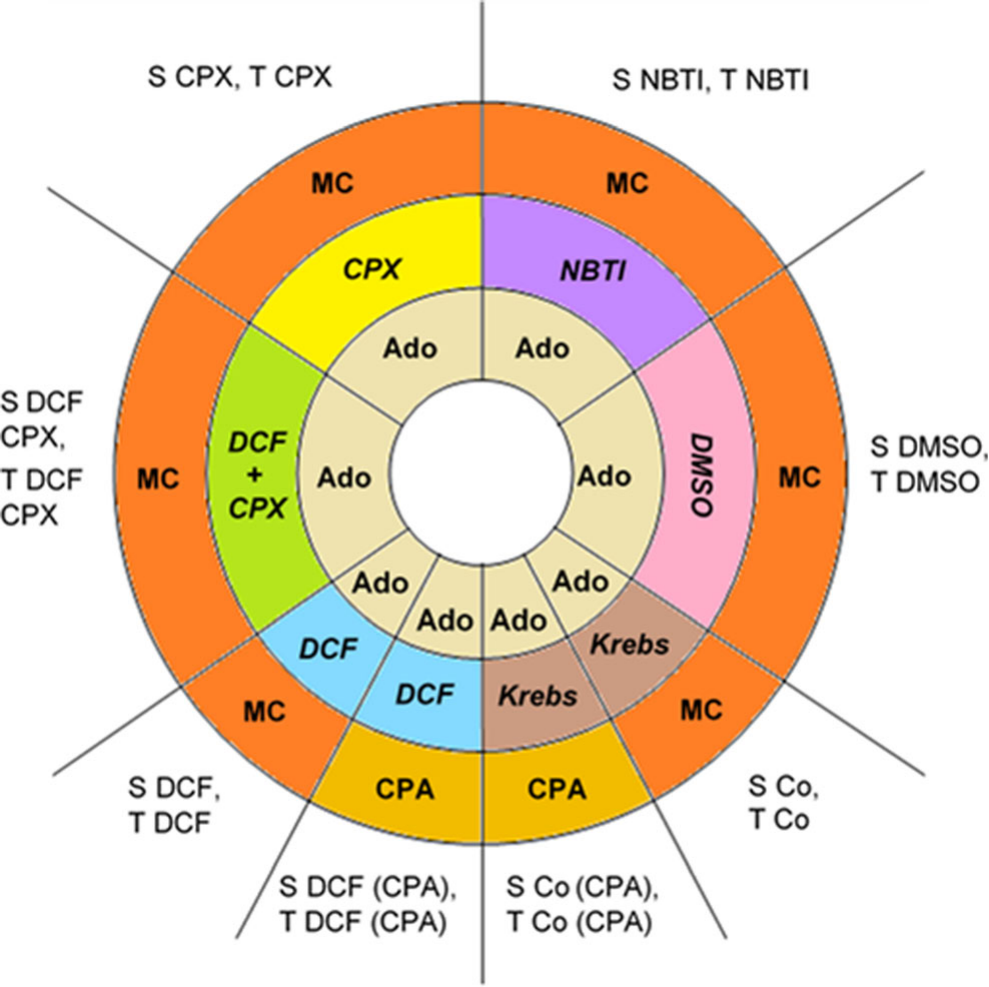
Conspectus of protocols and groups. The pivot of all protocols was the construction of two *E*/*c* curves with an in vitro treatment inserted into them. The *inner* and *outer annuli* of the pie chart represent the *E*/*c* curves (showing the agonist used), and the medium annulus symbolizes the in vitro treatment (indicating the applied chemicals in *italic*). *Sectors* of the pie chart denote the particular protocols that progressed from the inside out. The names of the experimental groups, which underwent the protocols, are listed *outside* of the sectors. Two groups belong to one protocol (one sector): one group including solvent-treated atria and another one involving T_4_-treated atria. *Ado* adenosine, *CPA* N^6^-cyclopentyladenosine, *MC* methacholine, *S* solvent treatment, *T* T_4_ (thyroxine) treatment, *Co* control, *Krebs* Krebs solution, *CPX* 8-cyclopentyl-1,3-dipropylxanthine, *DMSO* dimethyl-sulfoxide, *NBTI* nitrobenzylthioinosine, *DCF* 2′-deoxycoformycin

The in vitro treatment included 20 min of incubation in the presence of Krebs solution alone (S Co, T Co, S Co (CPA), T Co (CPA)) or 10 μM CPX (S CPX, T CPX) or 0.1 % (*v*/*v*) DMSO alone (S DMSO, T DMSO) or 10 μM NBTI (S NBTI, T NBTI) or 10 μM DCF (S DCF, T DCF, S DCF (CPA), T DCF (CPA)) or 10 μM DCF with 10 μM CPX (S DCF CPX, T DCF CPX). Finally, a cumulative *E*/*c* curve was generated with MC (S Co, T Co, S CPX, T CPX, S DMSO, T DMSO, S NBTI, T NBTI, S DCF, T DCF, S DCF CPX, T DCF CPX) or CPA (S Co (CPA), T Co (CPA), S DCF (CPA), T DCF (CPA)) (Fig. 1).

### Rationale for the experimental design

In the present study, the direct negative inotropic function was assessed because it proved to be a strong, well-measurable, and well-reproducible effect. Measurement was carried out on isolated and paced left atria, a greatly simplified model, in which the negative tropic effects of different agonists could manifest only in a decrease of the contractile force. This condition was important for the acquisition of accurate data because the direct component of negative inotropy is very sensitive to any change in the frequency of contraction (Endoh 1999). The reliability of the raw data was essential as opportunities inherent in RRM could be only exploited using accurate input data.

Protocols of the present study consist of the construction of two *E*/*c* curves and an in vitro treatment between them. For the first *E*/*c* curve, adenosine was used to assess the responsiveness of the naïve (in vitro untreated) atrial A_1_ receptors. Adenosine is especially suitable for this purpose because of its rapid elimination without yielding confounding byproducts (Wilbur and Marchlinski 1997). For the second *E*/*c* curve, MC or CPA (two relatively stable agonists for the M_2_ or A_1_ receptor, respectively) was administered in order to gather information about the effect of the different in vitro treatments on the M_2_ and A_1_ receptor responses. In the atrium, signaling pathways of these two receptors are almost the same concerning the direct negative inotropic effect (Belardinelli et al. 1995; Harvey and Belevych 2003).

According to the major goal of this study, the ADA inhibitor DCF was administered during the in vitro treatment to investigate the effects of ADA inhibition on the atrial A_1_ adenosinergic system (which has a significant overlap with the M_2_ muscarinergic machinery). The A_1_ receptor antagonist CPX was added solely (to check its effect alone) and together with DCF (to explore the contribution of A_1_ receptors, activated by endogenous adenosine evading the deamination by ADA, to the effect of DCF on the MC *E*/*c* curve). Blockade of the inward adenosine transport elicited by NBTI was applied to generate data about the increase in concentration of interstitial adenosine in this particular experimental setup (using MC) and thereby to enable the comparison between ADA inhibition (by DCF) and nucleoside transport blockade (by NBTI) regarding their effect on the interstitial adenosine level. DMSO treatment served as control for the NBTI treatment (Fig. 1).

### Characterization of *E*/*c* curves

The effect (defined as a percentage decrease in the initial contractile force) obtained from the experiments was plotted against the concentration of agonists administered. Both individual and averaged *E*/*c* curves were fitted to the Hill equation (Hill 1910):

1 $$ E={E}_{\max}\cdot \frac{c^n}{c^n+{{\mathrm{EC}}_{50}}^n} $$

where *c* is the concentration of the agonist administered, *E* is the effect, *E*_max_ is the maximal effect, EC_50_ is the agonist concentration producing a half-maximal effect, and *n* is the Hill coefficient.

Hill parameters (*E*_max_, EC_50_, *n*) of the individual *E*/*c* curves were used for statistical analysis. Hill parameters of some averaged *E*/*c* curves were applied for the mathematical correction of some other *E*/*c* curves (see below).

### Comparison of *E*/*c* curves

The *E*/*c* curves were compared with each other in two manners: by means of their effect values at each concentration and per their Hill parameters. Three kinds of comparison were made: (1) Within the same in vivo treated atria, the adenosine *E*/*c* curves of different groups were compared with one another. Similarly, the MC *E*/*c* curves of the control-type groups (Co, CPX, DMSO) were also compared with one another (within the same in vivo treatment); (2) The groups receiving DCF and NBTI treatment were compared to their (same in vivo treated) control counterparts; (3) The pooled adenosine *E*/*c* curves of all T_4_-treated atria. Furthermore, the MC and CPA *E*/*c* curves of some T_4_-treated control groups (T Co, T DMSO, T Co (CPA)) were compared to their solvent-treated counterparts.

### Quantification of the *E*/*c* curve change caused by NBTI and DCF

The surplus interstitial adenosine, accumulated over the basal level in response to the inhibition of nucleoside transport or ADA, modified (“biased”) the shape of the *E*/*c* curves constructed in the presence of NBTI or DCF. As DCF was found previously to influence the signaling efficiency of atrial A_1_ adenosinergic system (Kemeny-Beke et al. 2007), the modified *E*/*c* curves generated with the A_1_ receptor agonist CPA were excluded from the quantification (i.e., CPA *E*/*c* curves of groups S Co (CPA), T Co (CPA), S DCF (CPA), and T DCF (CPA)). The transformation of *E*/*c* curves constructed with MC was quantified with the use of RRM (Gesztelyi et al. 2004; Grenczer et al. 2010a) by fitting the averaged data of the modified *E*/*c* curves to the following equation:

2 $$ E^{\prime }=100-\frac{100\cdot \left(100-{E}_{\max}\cdot \frac{{\left({c}_x+c\right)}^n}{{\left({c}_x+c\right)}^n+{{\mathrm{EC}}_{50}}^n}\right)}{100-{E}_{\max}\cdot \frac{{c_x}^n}{{c_x}^n+{{\mathrm{EC}}_{50}}^n}} $$

where *E*′ is the averaged effect value of the modified MC *E*/*c* curve of the group S NBTI, T NBTI, S DCF, or T DCF, *E*_max_, EC_50_, *n* is the Hill parameters of the corresponding control-type averaged MC *E*/*c* curve of the group S DMSO, T DMSO, S Co, or T Co, respectively, *c* is the concentration of MC administered for the *E*/*c* curve, and *c*_*x*_ is the MC concentration that is equieffective with the surplus interstitial adenosine accumulated by NBTI or DCF.

### Correction of effect values for the change produced by NBTI and DCF

The correction procedure was performed as described previously (Kiss et al. 2013). First, an effect belonging to *c*_*x*_ was calculated by means of the Hill equation:

3 $$ {E}_x={E}_{\max}\cdot \frac{{c_x}^n}{{c_x}^n+{{\mathrm{EC}}_{50}}^n} $$

where *E*_*x*_ is the effect evoked solely by the surplus interstitial adenosine produced by NBTI or DCF, *c*_*x*_ is the MC concentration conveyed by Eq. 2 (belonging to the averaged MC *E*/*c* curve of the group S NBTI, T NBTI, S DCF, or T DCF), *E*_max_, EC_50_, *n* is the Hill parameters of the corresponding control-type *E*/*c* curve (i.e., the averaged MC *E*/*c* curve of the group S DMSO, T DMSO, S Co, or T Co, respectively).

Then, from effect values of a modified *E*/*c* curve (*E*′) and the corresponding *E*_*x*_, corrected effect values were computed with the use of the following equation:

4 $$ E=100-\frac{\left(100-E^{\prime}\right)\cdot \left(100-{E}_x\right)}{100} $$

where *E* is the corrected effect, *E*′ is the modified effect, and *E*_*x*_ is the effect of the surplus interstitial adenosine produced by NBTI or DCF (belonging to the averaged MC *E*/*c* curve of the group S NBTI, T NBTI, S DCF, or T DCF).

In order to correct the effect values of MC *E*/*c* curves of groups S NBTI, T NBTI, S DCF, and T DCF, the averaged modified effects of these *E*/*c* curves (as *E*′) and the corresponding *E*_*x*_ values were substituted into Eq. 4. For the correction of CPA *E*/*c* curves of groups S DCF (CPA) and T DCF (CPA), the averaged modified effects of these *E*/*c* curves were substituted into Eq. 4 along with *E*_*x*_ values belonging to the averaged MC *E*/*c* curves of groups S DCF and T DCF, respectively. The reason of doing this was that the amount and effect of the surplus interstitial adenosine produced by DCF did not depend on the nature of the agonist used for a subsequent *E*/*c* curve. All corrected effects were plotted versus the MC and CPA concentrations administered for the given *E*/*c* curve.

### Data analysis and presentation

Each atrium was required to meet three criteria in order to qualify for inclusion in the statistical analysis: (1) the initial contractile force had to reach 1 mN before the first *E*/*c* curve; (2) the mechanical activity of the paced atrium had to be regular; and (3) the response to 10 or 100 μM adenosine of the solvent- or T_4_-treated atrium, respectively, was required to be within a mean ± 2 SD range. The mean and SD were computed using atria meeting the first two criteria (separately for the solvent- and T_4_-treated population). All experimental outcomes conforming to these three criteria were subjected to statistical workup.

According to the recommendation of Motulsky and Christopoulos (2004), concentrations in the equations used for curve fitting (*c*, EC_50_, and *c*_*x*_) were expressed as common logarithms.

Hill parameters of the pooled adenosine *E*/*c* curves (solvent-treated atria vs. T_4_-treated ones) and raw *E*/*c* data of selected *E*/*c* curve pairs were compared with unpaired Student *t* test or *t* test with Welch’s correction (if equal variance test was not passed) or Mann–Whitney *U*-test (if either equal variance test or normality test was not passed). Hill parameters of adenosine *E*/*c* curves of the different groups were compared (separately for the solvent and T_4_ treatment) by one-way ANOVA (using Geisser–Greenhouse correction) with Tukey post-testing or by Kruskal–Wallis test with Dunn’s post-testing (if the normality test was not passed). Hill parameters of the MC and CPA *E*/*c* curves were compared using two-way ANOVA with Sidak post-testing (as all data sets passed the normality test). Statistical significance for the difference of means (or medians) was assigned into one of four categories: *p* > 0.05 (not significant), *p* < 0.05 (one mark), *p* < 0.01 (two marks), or *p* < 0.001 (three marks).

Data presented in this paper are expressed as mean ± SEM or value with lower and upper 95 % confidence interval limits. Curve fitting and statistical analysis were performed with the use of GraphPad Prism 6.05, while other calculations were made by means of Microsoft Office Excel 2013.

## Results

### Thyroid state

By the ninth day, body weight of the solvent- and T_4_-treated guinea pigs were altered from 712 ± 11 to 723 ± 11 g and from 719 ± 12 to 538 ± 11 g (*p* < 0.001), respectively. The ratio of left atrial weight to body weight in the solvent- and T_4_-treated group was 0.089 ± 0.009 and 0.132 ± 0.009 mg/g (*p* < 0.001), respectively.

### Initial contractile forces

**Fig. 2:**
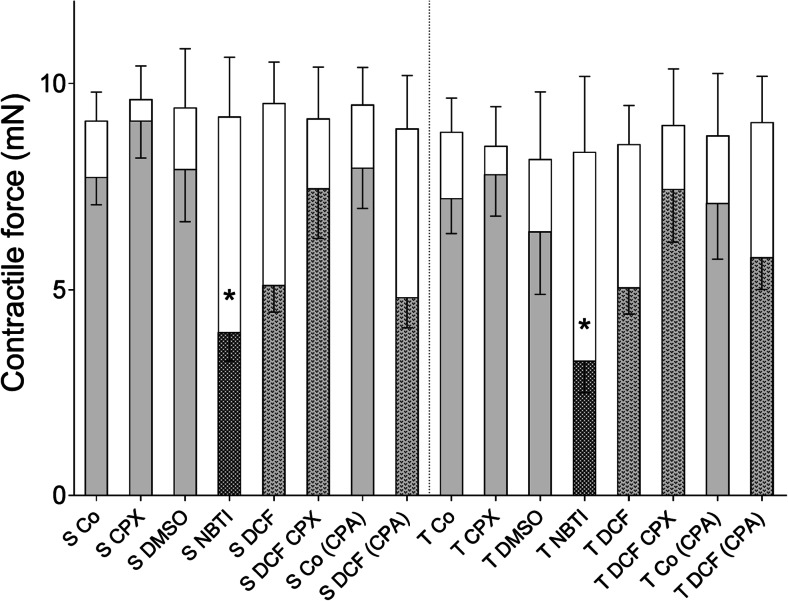
The contractile force of guinea pig left atria in the different groups before and after the in vitro treatment. The *open columns* show the contractile forces of atria measured before the in vitro treatment (mean + SEM). The *shorter filled columns* in front of the open ones denote the contractile forces determined at the end of the in vitro treatment (mean − SEM). The *homogeneous light grey filling* means an in vitro treatment containing neither NBTI nor DCF, while *light grey filling with darker or less dark pattern* shows an in vitro treatment containing NBTI or DCF, respectively. Group names are indicated *below* the columns. Comparing the contractile forces of the different in vitro-treated groups (filled columns), a significant difference was found only in the case of NBTI: S Co vs. S NBTI, S CPX vs. S NBTI, T Co vs. T NBTI, and T CPX vs. T NBTI (*asterisk*)

The initial contractile forces, measured prior to the in vitro treatment (before the first *E*/*c* curve), did not differ significantly either between the pooled solvent- and T_4_-treated atria (9.3 ± 0.35 and 8.67 ± 0.4 mN, respectively) or among the different groups (Fig. 2).

During the in vitro treatment, CPX non-significantly moderated the small decay in the contractile force over time as compared to the corresponding controls. In contrast, both NBTI and DCF decreased the atrial contractile forces, although this effect was significant only in the case of NBTI (*p* < 0.05). The co-treatment with DCF and CPX produced an outcome similar to the corresponding controls; thus, DCF and CPX appeared to mutually cancel out each other’s effects. Contractile forces of the different T_4_-treated groups did not differ significantly from their solvent-treated counterparts (Fig. 2).

### Adenosine *E*/*c* curves

#### Response to adenosine

**Fig. 3:**
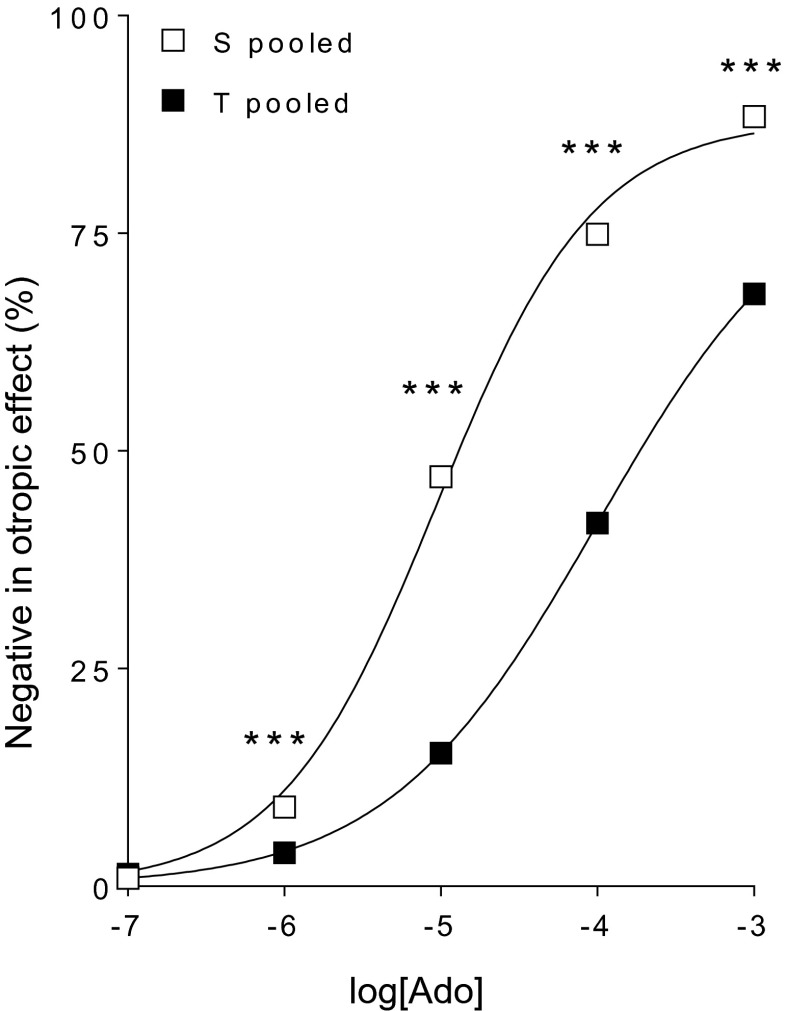
The direct negative inotropic effect of adenosine (*Ado*) in solvent-treated (*S*; *open symbols*) or T_4_-treated (*T*; *filled symbols*) guinea pig left atria (before any in vitro treatment). The *x-axis* shows the common logarithm of the molar concentration of adenosine, and the *y-axis* denotes the effect as a percentage decrease of the initial contractile force. The *symbols* indicate the responses to adenosine that were averaged after pooling the same in vivo-treated groups (± SEM). The *curves* illustrate the fitted Hill equation (Eq. 1). The responses to adenosine differed significantly between the pooled groups (*asterisk*)

Adenosine concentration dependently reduced the contractile force of atria (Fig. 3). As no previous intervention evoking positive inotropic effect was done, the response to adenosine was considered direct negative inotropic effect that is typical for the atrial (but not ventricular) myocardium (Belardinelli et al. 1995; Kurachi 1995).

#### Comparison before the in vitro treatment

Responses to the different adenosine concentrations (data not shown) as well as Hill parameters of the adenosine *E*/*c* curves (Table 1) did not differ significantly when compared among the same in vivo-treated groups with one another. Thus, the solvent- and T_4_-treated atria formed two homogenous populations regarding the response to adenosine.

**Table 1:** Hill parameters of the first *E*/*c* curve generated with adenosine (before the in vitro treatment indicated in the group names)

Ado curves	*E*_max_	logEC_50_	*n* (mean ± SEM)
S-pooled	88.27 ± 0.65	−5 ± 0.03	0.9 ± 0.03
T-pooled	84.53 ± 0.84**	−3.96 ± 0.03***	0.66 ± 0.01***
S Co	89.76 ± 1.24	−4.99 ± 0.06	0.8 ± 0.05
S CPX	87.42 ± 1.7	−4.94 ± 0.1	0.93 ± 0.07
S DMSO	87.59 ± 1.58	−5.03 ± 0.1	0.94 ± 0.08
S NBTI	89.59 ± 3.05	−4.9 ± 0.08	0.97 ± 0.15
S DCF	88.89 ± 0.62	−5.12 ± 0.02	0.91 ± 0.03
S DCF CPX	88.04 ± 1.03	−5 ± 0.06	0.88 ± 0.04
S Co (CPA)	87.78 ± 1.41	−5.05 ± 0.04	0.94 ± 0.04
S DCF (CPA)	90.79 ± 0.91	−5 ± 0.08	0.94 ± 0.06
T Co	85.81 ± 1.91	−3.9 ± 0.06	0.63 ± 0.02
T CPX	82.92 ± 1.96	−4 ± 0.1	0.72 ± 0.05
T DMSO	81.89 ± 2.4	−3.97 ± 0.1	0.72 ± 0.04
T NBTI	82.35 ± 2.89	−4.05 ± 0.09	0.67 ± 0.03
T DCF	86.05 ± 2.29	−3.88 ± 0.06	0.62 ± 0.03
T DCF CPX	85.73 ± 2.82	−3.92 ± 0.06	0.66 ± 0.04
T Co (CPA)	83.92 ± 2.59	−4.08 ± 0.08	0.75 ± 0.05
T DCF (CPA)	82.06 ± 2.67	−4.06 ± 0.11	0.74 ± 0.04

*E*_max_, logEC_50_ and *n* (mean ± SEM) are best-fit values of the Hill equation (Eq. 1) fitted to the individual adenosine *E*/*c* curves. Comparison was made between the pooled solvent (S)- and pooled T_4_ (T)-treated groups, furthermore within the same in vivo treated groups. Significant differences were found between the pooled groups (*asterisk*)

#### Effect of T_4_ on the response to adenosine

The responses to adenosine at the different concentrations (except the starting 0.1 μM) showed a significant decrease when comparing the pooled T_4_-treated atria to the pooled solvent-treated ones (Fig. 3). Consistently, the T_4_ treatment diminished *E*_max_, increased logEC_50_, and decreased *n* of these pooled adenosine *E*/*c* curves in a statistically significant manner (Table 1).

### MC *E*/*c* curves

#### Response to MC

**Fig. 4:**
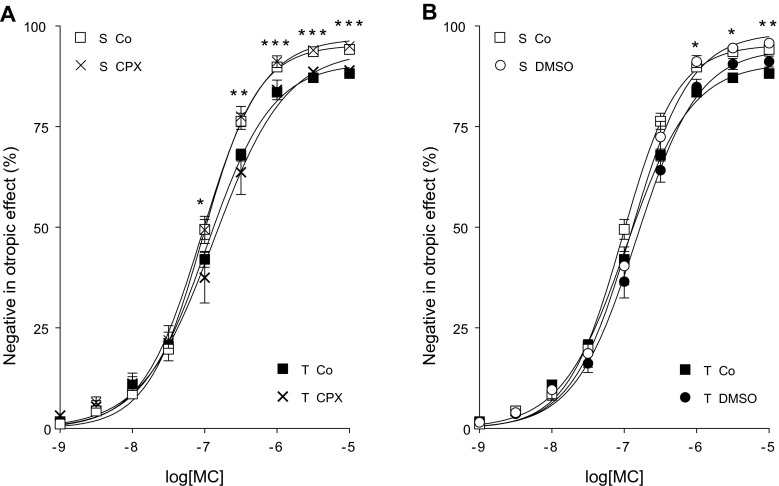
**a**, **b** The direct negative inotropic effect of MC in the presence of CPX or DMSO (alone) or in the absence of both of them (*Co*) in solvent-treated (*open*/*thin symbols*) or T_4_-treated (*filled*/*thick symbols*) guinea pig left atria. The *x-axis* shows the common logarithm of the molar concentration of MC, while the *y-axis* indicates the effect as a percentage decrease of the initial contractile force of atria. The *symbols* represent the responses to MC averaged within the groups (± SEM), and the *curves* illustrate the fitted Hill equation (Eq. 1). The responses to MC differed significantly between groups S Co vs. T Co and S DMSO vs. T DMSO (*asterisk*)

**Fig. 5:**
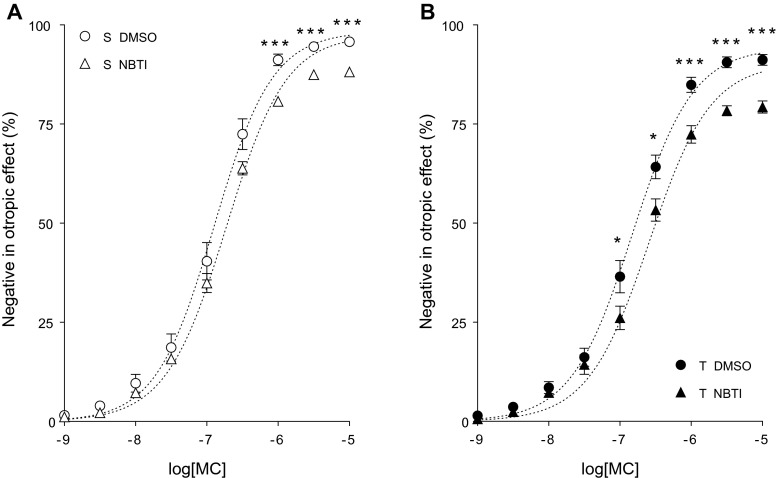
The direct negative inotropic effect of MC in the presence of DMSO (alone) or NBTI in solvent-treated (*open symbols* in **a**) or T_4_-treated (*filled symbols* in **b**) guinea pig left atria. The *x-axis* shows the common logarithm of the molar concentration of MC, and the *y-axis* indicates the effect as a percentage decrease of the initial contractile force of atria. The *symbols* denote the responses to MC averaged within the groups (± SEM), and the *dotted curves* illustrate the fitted RRM model (Eq. 2). The responses to MC differed significantly between groups S DMSO vs. S NBTI and T DMSO vs. T NBTI (*asterisk*)

**Fig. 6:**
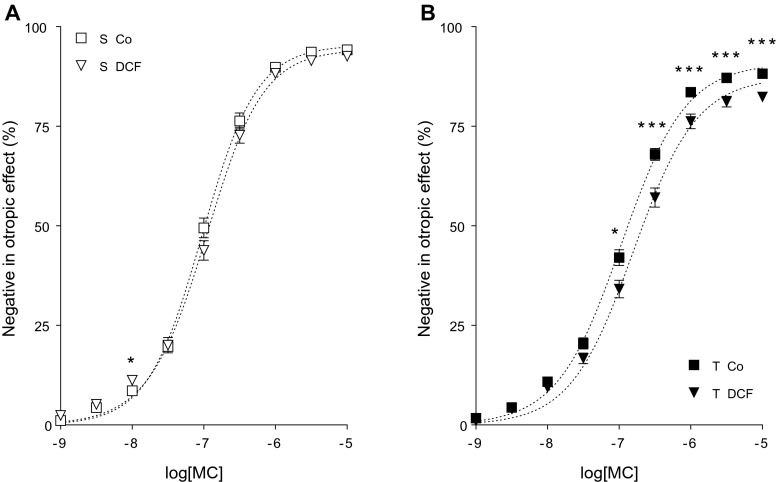
The direct negative inotropic effect of MC in the absence or presence of DCF in solvent-treated (*open symbols* in **a**) or T_4_-treated (*filled symbols* in **b**) guinea pig left atria. The *x-axis* denotes the common logarithm of the molar concentration of MC, and the *y-axis* indicates the effect as a percentage decrease of the initial contractile force of atria. The *symbols* show the responses to MC averaged within the groups (± SEM), and the *dotted curves* illustrate the fitted RRM model (Eq. 2). The responses to MC differed significantly between groups S Co vs. S DCF and T Co vs. T DCF (*asterisk*)

**Fig. 7:**
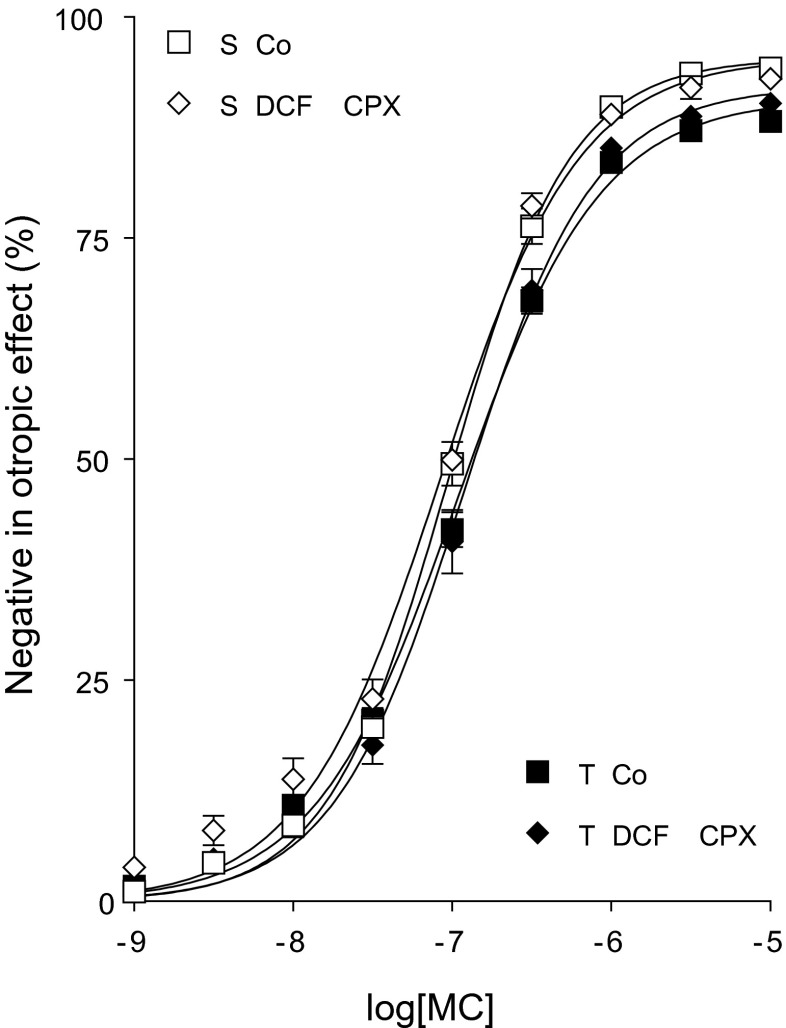
The direct negative inotropic effect of MC in the absence or presence of DCF added together with CPX in solvent-treated (*open symbols*) or T_4_-treated (*filled symbols*) guinea pig left atria. The *x-axis* denotes the common logarithm of the molar concentration of MC, and the *y-axis* indicates the effect as a percentage decrease of the initial contractile force of atria. The *symbols* show the responses to MC averaged within the groups (± SEM), and the *curves* illustrate the fitted Hill equation (Eq. 1)

MC also decreased the contractile force of atria in a concentration-dependent manner (direct negative inotropic effect) (Figs. 4, 5, 6, and 7).

#### Effect of CPX and DMSO on the response to MC

The control-type groups (Co, CPX, DMSO) receiving the same in vivo treatment did not differ significantly from one another when either the responses to the different MC concentrations (Fig. 4) or the Hill parameters of the MC *E*/*c* curves (data not shown) were compared. This observation indicates that DMSO, vehicle of CPX and NBTI, and CPX did not influence significantly the response to MC. Nevertheless, a minor difference can be shown between groups S Co vs. S DMSO and T Co vs. T DMSO (Fig. 4b). Based on prior experiences of our team with DMSO, this cannot be ascribed to the effect of DMSO but, rather, to the fact that investigations using NBTI (including their controls) were performed after experiments using CPX and DCF (and their controls). Seasonal differences can affect the atrial inotropic response (Kemeny-Beke et al. 2007).

#### Effect of T_4_ on the response to MC

Based on the comparison of groups T Co and T DMSO to their solvent-treated counterparts (S Co and S DMSO), the T_4_ treatment moderately suppressed the response to MC that was only significant at higher MC concentrations (Fig. 4). In line with this, the T_4_ treatment caused a moderate diminution in *E*_max_ (significant) and *n* (on the border of statistical significance), while the increase of logEC_50_ did not reach the significance threshold (Table 2).

**Table 2:** The Hill parameters exhibiting statistically significant change in response to the different in vivo (*asterisk*) or in vitro (*not-equal-to symbol*) treatments

	*E*_max_	logEC_50_	*n* (mean ± SEM)
MC curves			
S Co	95.5 ± 0.41		1.18 ± 0.03
S DMSO	97.52 ± 0.81		
S NBTI	90.23 ± 0.75≠≠		
T Co	90.77 ± 0.79**		1 ± 0.03*
T DCF	86.23 ± 1.43**≠**		
T DMSO	94.1 ± 1.44*		
T NBTI	82.85 ± 2.11**≠ ≠ ≠**		
CPA curves			
S Co (CPA)	90.9 ± 1.59	−7.86 ± 0.08	1.06 ± 0.05
T Co (CPA)	84.04 ± 1.01**	−7.5 ± 0.06**	0.7 ± 0.04***
T DCF (CPA)		−7.77 ± 0.08**≠**	

*E*_max_, logEC_50_ and *n* (mean ± SEM) are best-fit values of the Hill equation (Eq. 1) fitted to the individual *E*/*c* curves generated with MC or CPA. Significant differences were found between groups S Co vs. T Co, S DMSO vs. T DMSO, and S Co (CPA) vs. T Co (CPA) (*asterisk*), furthermore with S DMSO vs. S NBTI, T DMSO vs. T NBTI, T Co vs. T DCF, and T Co (CPA) vs. T DCF (CPA) (*not-equal-to symbol*)

#### Modification of the response to MC by NBTI

In both the solvent- and T_4_-treated groups, NBTI significantly reduced the response to MC according to the conventionally plotted (and thereby modified) *E*/*c* curves (Fig. 5). This manifested in a significant decrease of *E*_max_ and in a minor increase of logEC_50_ with a practically unchanged *n* (Table 2). The effect of NBTI was more intense in the group T NBTI than in the group S NBTI (Fig. 5; Table 2).

#### Effect of NBTI on the interstitial adenosine level

Based on the depression of the conventionally plotted MC *E*/*c* curves generated in the presence of NBTI (Fig. 5), the surplus interstitial adenosine was found to be equieffective with 101.2 and 151.1 nM MC in the solvent- and T_4_-treated atria, respectively (Table 3). It means that nucleoside transport blockade produces a greater interstitial adenosine accumulation in the T_4_-treated atria than in the solvent-treated ones, consistent with our earlier studies in which CPA served as an agonist for the *E*/*c* curves (Karsai et al. 2007; Pak et al. 2014).

**Table 3:** The *c*_*x*_ values obtained with RRM characterizing the concentration of the surplus interstitial adenosine produced by NBTI or DCF in solvent (S)- or T_4_ (T)-treated guinea pig left atria

	Log*c*_*x*_	95% CI		*c*_*x*_ (nmol/l)
		From	To	
S DMSO	−12.62	−18,000	17,975	0.00024
S NBTI	−6.99	−7.1	−6.88	*101.2*
T DMSO	−9.07	−17.62	−0.53	0.84
T NBTI	−6.82	−6.93	−6.72	*151.1*
S Co	−13.98	−91,603	91,576	0.00001
S DCF	−7.55	−7.73	−7.37	*28.05*
T Co	−53.25	Ambiguous		0
T DCF	−7.35	−7.45	−7.26	*44.36*

The log*c*_*x*_ is the best-fit value of Eq. 2 fitted to the averaged *E*/*c* curves generated with MC in the presence of DMSO or NBTI or DCF or in the absence of all of them (Co). Precision of the fit was characterized with the 95 % confidence interval (95 % CI) for the best-fit value. Fitting of the control-type groups (S DMSO, T DMSO, S Co, T Co) to Eq. 2 served only for verification; the expected value of *c*_*x*_ was 0. In turn, fitting of the groups S NBTI, T NBTI, S DCF, and T DCF was expected to provide a *c*_*x*_ value (shown in *italic*) that indicates the MC concentration equieffective with the surplus interstitial adenosine produced by NBTI or DCF

#### Modification of the response to MC by DCF

DCF decreased the response to MC in both the solvent- and T_4_-treated atria according to the conventionally plotted (modified) *E*/*c* curves. This modification was rather symbolic under euthyroid conditions (Fig. 6a), while it was well marked in hyperthyroidism (Fig. 6b). In turn, CPX abolished this effect of DCF on the MC *E*/*c* curves in both thyroid states (Fig. 7). These observations indicate that DCF exerted its effect on the response to MC the same way as NBTI did, i.e., via elevating the interstitial adenosine level. It should be noted that the effect of DCF on the response to MC was statistically significant only in the T_4_-treated atria (except for the response at 10 nM MC in the group S DCF, but it was considered irrelevant) (Fig. 6). Consistently, the decrease of *E*_max_ was only significant in the group T DCF (Table 2).

#### Effect of DCF on the interstitial adenosine level

Fitting Eq. 2 to MC *E*/*c* data of groups S DCF and T DCF (Fig. 6), the surplus interstitial adenosine proved equieffective with 28.05 and 44.36 nM MC in the solvent- and T_4_-treated atria, respectively (Table 3). This outcome is similar to that seen in response to NBTI (Table 3), namely, DCF appears to produce a greater interstitial adenosine accumulation in the T_4_-treated atria than in the solvent-treated ones (Table 3). However, due to reasons specified in “Discussion”, this latter finding should be treated with caution. Nevertheless, DCF increased the interstitial adenosine level to a smaller extent than NBTI did in both the solvent- and T_4_-treated groups (Table 3).

### CPA *E*/*c* curves

#### Response to CPA

**Fig. 8:**
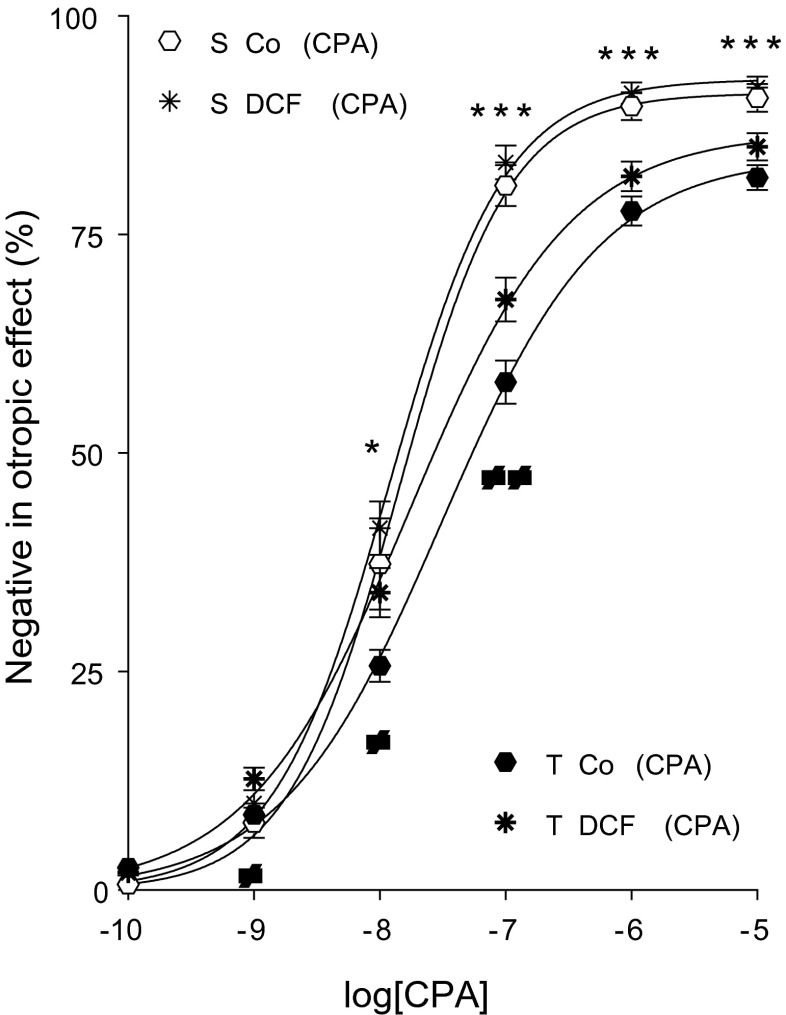
The direct negative inotropic effect of CPA in the absence or presence of DCF in solvent-treated (*open*/*thin symbols*) or T_4_-treated (*filled*/*thick symbols*) guinea pig left atria. The *x-axis* shows the common logarithm of the molar concentration of CPA, while the *y-axis* indicates the effect as a percentage decrease of the initial contractile force of atria. The *symbols* denote the responses to CPA averaged within the groups (± SEM), and the *curves* represent the fitted Hill equation (Eq. 1). The responses to CPA differed significantly between groups S Co (CPA) vs. T Co (CPA) (*asterisk*) and T Co (CPA) vs. T DCF (CPA) (*not-equal-to symbol*)

CPA also reduced the atrial contractile force in a concentration-dependent manner (direct negative inotropic effect) (Fig. 8).

#### Effect of T_4_ on the response to CPA

Comparing groups T Co (CPA) and S Co (CPA), the T_4_ treatment considerably decreased the response to CPA that was significant from medium to high concentrations (Fig. 8). In agreement with this, T_4_ treatment significantly reduced both *E*_max_ and *n* and increased logEC_50_ (Table 2). Thus, T_4_ induced a greater depression of the *E*/*c* curve for CPA than for MC (Figs. 4 and 8; Table 2).

#### Modification of the response to CPA by DCF

In contrast to that seen with MC (Fig. 6), DCF augmented the response to CPA in both the solvent- and T_4_-treated atria, according to the conventionally plotted (modified) *E*/*c* curves. While this effect of DCF was minor in the solvent-treated atria, it was significant in the T_4_-treated ones in the lower and medium concentration ranges (Fig. 8). In agreement with this, DCF significantly decreased logEC_50_ of *E*/*c* curves of T_4_-treated but not solvent-treated atria (Table 2). This outcome is consistent with our previous finding that inhibition of ADA enhances the efficiency of the direct negative inotropic function mediated by the A_1_ receptor in the hyperthyroid guinea pig atrium (Kemeny-Beke et al. 2007).

### Corrected MC and CPA *E*/*c* curves

The corrected *E*/*c* curves have two points of interest, the starting and final ones. The starting point shows *E*_*x*_, the effect belonging to *c*_*x*_, while the last point reflects the maximal response of the given system to the agonist in question. As *c*_*x*_ values have been addressed previously, herein the emphasis is on the final point of the corrected *E*/*c* curves, specifically on its position relative to the last point of the corresponding control *E*/*c* curve (the latter considered to be a priori correct).

#### NBTI with MC

**Fig. 9:**
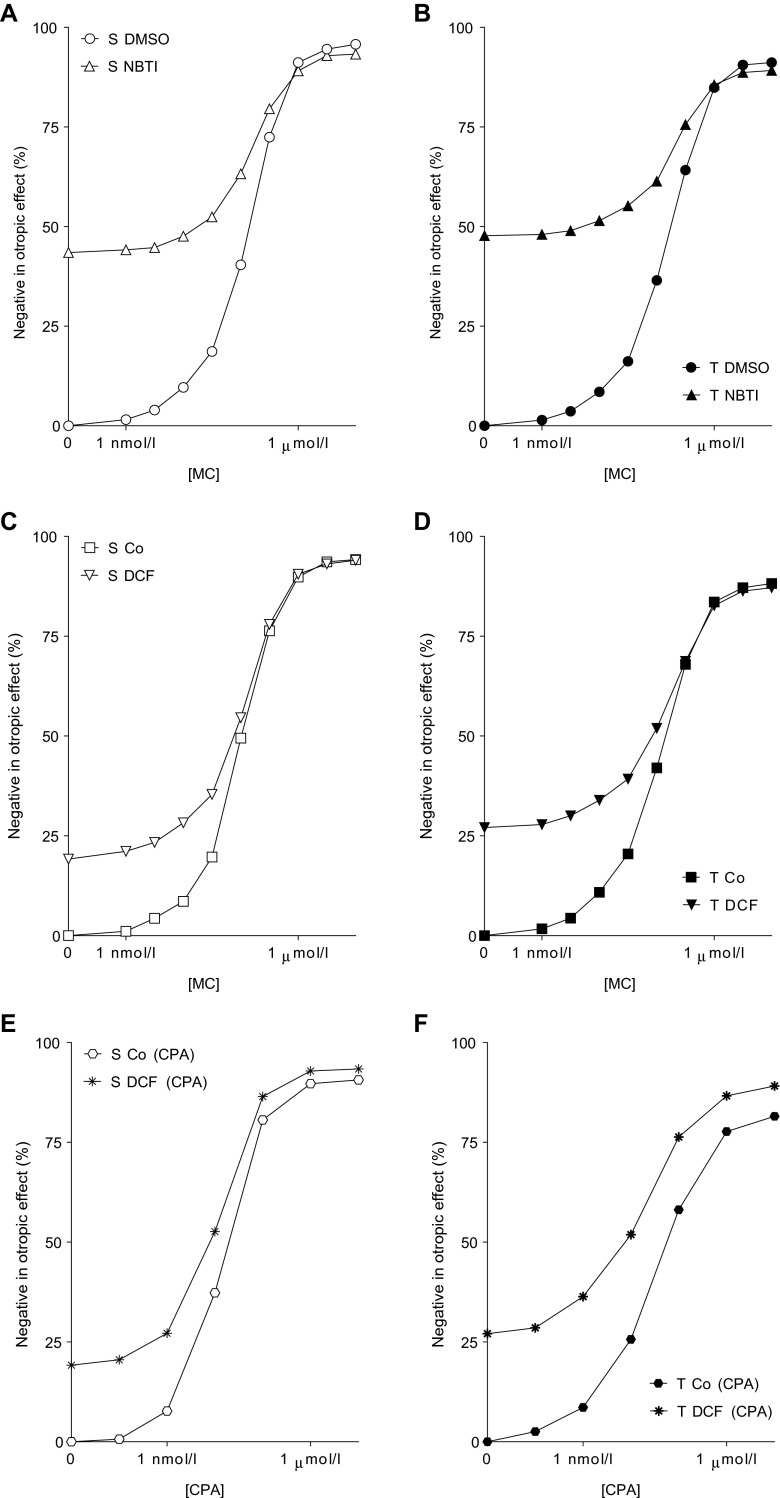
**a**–**f** The corrected direct negative inotropic effect values of concentration–effect curves generated in the presence of NBTI or DCF, plotted versus the concentration of the administered MC or CPA, together with the corresponding control concentration–effect curves (in their original form). The correction was made for the direct negative inotropic effect of the surplus interstitial adenosine produced by NBTI or DCF in a manner that this effect value has been incorporated into the effect data depicted herein. The *x-axis* indicates the agonist concentration on a base-10 logarithmic scale, and the *y-axis* shows the effect as a percentage decrease in the initial contractile force of atria. The *symbols* (*open*/*thin* solvent treatment, *filled*/*thick* T_4_ treatment) represent the corrected effects computed from the raw effects averaged within the groups

The corrected MC *E*/*c* curves (generated in the presence of NBTI) ended somewhat below their control curves (Fig. 9a, b). Thus, if we consider the observed small difference between maximal values of the corrected and control *E*/*c* curves to be an error, it can be concluded that NBTI does not affect the efficiency of the M_2_ muscarinergic signaling, irrespective of the thyroid state (Fig. 9a, b).

#### DCF with MC

The corrected MC *E*/*c* curves (constructed in the presence of DCF) ran to the maximum of their control curves (Fig. 9c, d). Conclusions to be drawn from this outcome are the same as those with NBTI, i.e., DCF does not influence the signal amplification of the atrial M_2_ muscarinergic mechanisms, regardless of the T_4_ treatment (Fig. 9c, d).

#### DCF with CPA

Effect values of the corrected CPA *E*/*c* curves (generated in the presence of DCF) exceeded their control effect values at each concentration, even at the highest one (Fig. 9e, f). This finding indicates that DCF augments the efficiency of the A_1_ adenosinergic system regarding its direct negative inotropic function in the guinea pig atrium. This phenomenon was visibly more pronounced in the T_4_-treated atria (Fig. 9e, f), in agreement with our previous finding (Kemeny-Beke et al. 2007). However, the present result denotes that DCF can exert its efficiency-enhancing effect even in the euthyroid state.

## Discussion

The first finding of the present study is that, in the guinea pig atrium, ADA inhibition (but not nucleoside transport blockade) enhances the efficiency of the direct negative inotropic function of the A_1_ adenosine (but not M_2_ muscarinic) receptor. This finding suggests that inhibition of ADA affects the atrial A_1_ adenosinergic system in a part other than the joint signaling pathways of the A_1_ and M_2_ receptors. Second, ADA inhibition enhances the A_1_ adenosinergic direct negative inotropy even in the euthyroid state, although to a less extent than in hyperthyroidism. This outcome implies that ADA inhibition can partially reset the A_1_ receptor-mediated direct negative inotropy suppressed by thyroid hormones. Third, ADA inhibition produces a smaller rise in the interstitial adenosine concentration than nucleoside transport blockade does. Fourth, our results demonstrate that T_4_ treatment suppresses the direct negative inotropic function of the M_2_ receptor in a guinea pig model as well. Nevertheless, this reduction in the M_2_ muscarinergic function in response to thyroid hormones is quite small relative to the decrease in the A_1_ adenosinergic one.

Previously we found that ADA inhibition elicited by DCF potentiated the direct negative inotropic effect of CPA, a selective A_1_ receptor agonist, in the hyperthyroid guinea pig atrium (Kemeny-Beke et al. 2007). Since CPA is not a substrate for ADA (Pavan and Ijzerman 1998), this counterintuitive result was attributed to the fact that ADA inhibition increases the signal amplification of the atrial A_1_ receptor and/or its downstream signaling pathways under hyperthyroid conditions. We assumed that this efficiency-enhancing effect of ADA inhibition may be associated with intracellular adenosine accumulation rather than the interstitial one (the latter of which is otherwise responsible for the stimulation of the cell surface A_1_ receptors, a known trigger of several beneficial effects of ADA inhibition; Zhu et al. 1994; Peart et al. 2001; Willems et al. 2006; Szentmiklosi et al. 2011). This assumption is supported by the fact that blockade of the physiologically inward nucleoside transport, which also increases the interstitial adenosine concentration but decreases the intracellular one (Deussen 2000a, 2000b), does not enhance the efficiency of the atrial A_1_ adenosinergic system under either euthyroid or hyperthyroid conditions (Karsai et al. 2006, 2007).

The major difficulty to investigate this phenomenon is the fact that ADA inhibition, besides enhancing the efficiency of A_1_ receptor function (an effect first described by Kemeny-Beke et al. in 2007), elevates the tissue adenosine content that leads to A_1_ receptor activation that also augments the A_1_ receptor-mediated functions (a well-known effect). In the experimental setup used for the above-mentioned study (Kemeny-Beke et al. 2007), this problem occurred in a form such that the decrease of the contractile force (evoked by the surplus interstitial adenosine caused by ADA inhibition) interfered with the efficiency-enhancing effect of ADA inhibition on the A_1_ receptor-mediated direct negative inotropy. To clarify this case, we provide a brief explanation. When DCF had been administered, the interstitial adenosine level increased, and the surplus adenosine exerted a direct negative inotropic effect on the atria. So, this condition served as baseline for further manipulations, i.e., administration of CPA to generate an *E*/*c* curve. As the surplus interstitial adenosine had already consumed a part of the response capacity of the A_1_ adenosinergic system, a modified (smaller than expected) response to CPA was detected (for more details about this phenomenon, see Gesztelyi et al. 2004; Grenczer et al. 2010a). Thus, the two actions of ADA inhibition (mentioned at the top of this paragraph) worked against each other in our earlier experimental setup (Kemeny-Beke et al. 2007).

In the present study, we aimed to separate these two actions of ADA inhibition in order to gain a deeper understanding of the influence of ADA inhibition on the regulation of contractility of the atrium. To address this challenge, we repeated our previous experiments with the replacement of CPA with MC, a muscarinic receptor agonist with high affinity for the M_2_ receptor.

In the heart, the A_1_ and M_2_ receptors are the predominant receptor types for adenosine (Headrick et al. 2013; Ijzerman et al. 2014) and acetylcholine (Harvey 2012; Birdsall et al. 2014), respectively. In the atrium, both A_1_ and M_2_ receptors bind to G_i/0_ proteins, and thereby both of them can open the muscarinic-operated potassium channel and blunt the adenylyl cyclase activity with the consequent inhibition of all cAMP-dependent signaling pathways (Caulfield and Birdsall 1998; Fredholm et al. 2001, 2011; Harvey and Belevych 2003; Harvey 2012; Headrick et al. 2011, 2013). As a consequence, both A_1_ and M_2_ receptors can mediate a direct negative inotropic effect (Belardinelli et al. 1995; Kurachi 1995). Thus, although via binding to different receptors, CPA and MC activate greatly overlapping signal transduction pathways in the atrium.

The key concept of the experimental design used for the present study is as follows: If the A_1_ receptor and ADA are inhibited simultaneously, the direct negative inotropic effect of the surplus interstitial adenosine produced by ADA inhibition can be prevented (with the consequent preservation of the response capacity of the A_1_ adenosinergic system). However, the major signaling pathways underlying the direct negative inotropy remain accessible from the M_2_ receptor. In this setup, if the molecular target, the change of which is responsible for the enhanced efficiency of the direct negative inotropic function of the A_1_ receptor under ADA inhibition, is located in the joint part of the postreceptorial signaling of A_1_ and M_2_ receptors, an enhanced response to MC is expected relative to the naïve state (lacking A_1_ receptor antagonist and ADA inhibitor). If this is the case, we succeed in narrowing the circle of possible mechanisms of action for the efficiency-enhancing effect of ADA inhibition. If not, besides narrowing the circle of possible action mechanisms (i.e., the molecular target in question is out of the shared part of signaling of the A_1_ and M_2_ receptors), we have the opportunity to quantify the concentration of the surplus interstitial adenosine produced by ADA inhibition. This is because a prerequisite of the quantification is the fixedness of signal amplification properties of the M_2_ muscarinergic system throughout the investigation.

Results of a preliminary study, carried out in our laboratory using a simple protocol and small sample size, suggested that ADA inhibition might enhance the response to MC (in addition to CPA) (Grenczer et al. 2007). During the present investigation, we have expanded our protocol with the use of CPX, a selective A_1_ receptor antagonist, and applied a sufficiently large sample size. Our current results show that, in the presence of CPX, ADA inhibition afforded by DCF was unable to influence the response to MC (Fig. 7). Thus, we have every reason to conclude that ADA inhibition does not affect the shared part of postreceptorial signaling of A_1_ and M_2_ receptors. Therefore, we could quantify the effect of ADA inhibition on the interstitial adenosine concentration and then compare it with the similar action of nucleoside transport blockade (by NBTI).

The response to MC during inhibition of the nucleoside transport or ADA (without A_1_ receptor blockade) showed a decrease relative to the naïve state. This phenomenon was due to the fact that the surplus interstitial adenosine, by activating the A_1_ adenosinergic machinery, modified (“biased”) the effect mediated by the M_2_ receptor. Namely, because of the overlapping signaling pathways, when a fraction of the response capacity of the A_1_ adenosinergic system was depleted, the responsiveness of the M_2_ muscarinergic system also decreased (Gesztelyi et al. 2004). The magnitude of the change (“bias”) of the *E*/*c* curve is characteristic of the magnitude of the modifying effect (Grenczer et al. 2010a).

To estimate the surplus interstitial adenosine from the modification of MC *E*/*c* curves, RRM presented itself. The motif of RRM is the interchangeability of agonists evoking the same kind of effect, irrespective of what sort of receptor they bind to (Gesztelyi et al. 2004; Grenczer et al. 2010a, 2010b). In the present case, RRM has quantified the surplus interstitial adenosine with a surrogate parameter, i.e., the equieffective MC concentration (*c*_*x*_).

The *c*_*x*_ values of the present study show that NBTI substantially elevated the interstitial adenosine level, and this effect was greater in the hyperthyroid guinea pig atrium than in the euthyroid one (Table 3). This finding corroborates our previous results about the effect of NBTI, in which *c*_*x*_ values were obtained as equieffective CPA concentrations (Karsai et al. 2006, 2007; Kiss et al. 2013; Pak et al. 2014).

The *c*_*x*_ values of the present study also indicate that DCF, similarly to NBTI, increased the interstitial adenosine level in both the eu- and hyperthyroid guinea pig atria. Expressing this action in numbers, NBTI produced an about 3.5-fold greater *c*_*x*_ than DCF did, irrespectively of the thyroid state (Table 3). This denotes that the nucleoside transport blockade has greater influence on the interstitial adenosine level than ADA inhibition (under our ex vivo conditions ensuring a well-oxygenated bathing medium). However, nucleoside transport inhibitors increases the interstitial adenosine level only in the metabolically intact myocardium (Deussen et al. 1999; Deussen 2000a, 2000b). In hypoxia, nucleoside transport blockers can decrease the interstitial adenosine level by inhibiting the adenosine release from the cells (Görge et al. 1998; Schreieck and Richardt 1999). Thus, to elevate the interstitial adenosine concentration, ADA inhibition appears to be an intervention more reliable than nucleoside transport blockade. It should be noted that ADA inhibitors have a wide range of actions throughout the body that forms the basis for several side effects (Bazl et al. 2012). However, these side effects are less problematic if ADA inhibitors are applied in an isolated organ rather than the whole body, e.g., in a heart to be transplanted. ADA inhibitors have been found to reduce hypoxic injury during cardiac surgery (Zhu et al. 1994; Hudspeth et al. 1994; Abd-Elfattah et al. 2013).

Because of unaltered signal amplification properties of the M_2_ muscarinergic system under ADA inhibition (Figs. 7 and 9c, d), *c*_*x*_ values obtained from MC *E*/*c* curves could be used to correct the conventionally plotted MC and CPA *E*/*c* curves generated in the presence of NBTI and DCF (without CPX) for the change produced by the surplus interstitial adenosine. As the exact concentration of extra adenosine at the A_1_ receptors remained unknown, the modified effect values were only corrected, and then they were plotted against the concentration of the agonist administered for the *E*/*c* curve. Therefore, the two most useful points of the corrected *E*/*c* curves are those at zero and at the highest concentration. The starting point shows the effect evoked by the surplus interstitial adenosine alone (*E*_*x*_), while the final one represents the maximal response of the given system to the given agonist (owing to the fact that well-saturated *E*/*c* curves were corrected).

The most important feature of the corrected MC *E*/*c* curves is that all of them end practically ibidem as their controls (considered to be inherently correct). This behavior of the curves confirms that the efficiency of the M_2_ muscarinergic control on atrial contractility did not change in response to the inhibition of either nucleoside transport or ADA (Fig. 9a–d).

In contrast, the corrected CPA *E*/*c* curves exceed their controls at the highest CPA concentration that behavior is especially conspicuous in the hyperthyroid atria (Fig. 9e, f). It can be concluded that ADA inhibition increases the efficiency of the A_1_ adenosinergic direct negative inotropic function, even in the euthyroid state. Nevertheless, consistent with our previous observation (Kemeny-Beke et al. 2007), this efficiency-enhancing effect of ADA inhibition is stronger in hyperthyroidism. Based on the comparison of the present results obtained using NBTI with those applying DCF, the efficiency-enhancing effect of ADA inhibition may be speculated to be induced by a rise in the intracellular rather than interstitial adenosine level.

Our present results denote that ADA inhibition readjusts the T_4_-induced suppression (Szentmiklosi et al. 1992; Kaasik et al. 1994) in the capacity of the A_1_ receptor-mediated direct negative inotropic function, an adenosinergic protective (energy consumption limiting) effect. The impact of this finding stems from the fact that excess thyroid hormones place an extra burden on the heart (Cini et al. 2009; Nabbout and Robbins 2010) and increase the risk of ischemic heart disease, supraventricular arrhythmias and congestive heart failure (Franklyn and Boelaert 2012). Thus, the enhancement of endogenous protective ability of the heart by means of ADA inhibition seems to be an especially promising possibility in hyperthyroidism.

However, an important (and until now overlooked) factor should be also taken into account concerning the quantification of surplus interstitial adenosine and the correction for the resulting *E*/*c* curve transformation: the change in the signal amplification of A_1_ adenosinergic system in response to ADA inhibition. This phenomenon could affect the assessment of *c*_*x*_ values under ADA inhibition, namely, it probably caused an overestimation of *c*_*x*_ values (Fig. 9c, d; Table 3). For explanation, the following should be thought over. If the A_1_ adenosinergic machinery is more sensitive (because of ADA inhibition), a given quantity of adenosine evokes a greater effect (*E*_*x*_) and thereby causes a larger change in the MC *E*/*c* curve. From this larger change, a greater *c*_*x*_ can be estimated by means of RRM (with the use of Hill parameters of the corresponding control MC *E*/*c* curve; see: Eq. 2). (Thus, *c*_*x*_ values determined under ADA inhibition are MC concentrations that are equieffective with the surplus interstitial adenosine if it activates an A_1_ adenosinergic system amplified by ADA inhibition.)

The overestimation of *c*_*x*_ values for ADA inhibition has two important consequences concerning our results. First, as the efficiency enhancing effect of ADA inhibition is stronger in the hyperthyroid atria, no conclusion is worth being drawn from the greater *c*_*x*_ in the presence of DCF in hyperthyroidism (Table 3). Second, this phenomenon also affects the comparison of *c*_*x*_ values reflecting the effect of NBTI with those of DCF. However, the former *c*_*x*_ values are even so greater than the latter (although overestimated) *c*_*x*_ values (Table 3); therefore, the conclusion drawn previously (i.e., NBTI is superior to DCF in increasing the interstitial adenosine level) has remained true (moreover, it has become even truer).

At the same time, it can be stated that the corrected *E*/*c* curves generated in the presence of DCF are even so appropriate. This is due to the fact that although *c*_*x*_ values determined under ADA inhibition are overestimated, they were used to correct *E*/*c* curves constructed under ADA inhibition. During the correction, *c*_*x*_ values were converted into modifying effects (*E*_*x*_) by means of the same Hill parameters that were used to obtain these *c*_*x*_ values. So, in the course of the correction, we got correct *E*_*x*_ values, and these effects were applied for further calculations. Thus, conclusions drawn from the corrected *E*/*c* curves are all valid.

In addition, to the best of our knowledge, this study is the first to report about the suppressing effect of T_4_ treatment on the inotropic control exerted by the atrial M_2_ muscarinic system in a guinea pig model. On the other hand, while thyroid hormones remarkably decreased *E*_max_ and the potency of the direct negative inotropic action of muscarinic agonists (MC and carbachol) in the rat left atrium (Ishac and Pennefather 1983), we found this effect to be quite small in the guinea pig left atrium, manifested in a moderate decrease in *E*_max_ and a minor reduction in the Hill coefficient (Fig. 4; Table 2).

The major limitation of the present study is the pure functional approach applied in it. This was necessary to unequivocally detect the nature and extent of the influence of ADA inhibition on the contractility of the working supraventricular myocardium. Of course, this does not substitute subsequent molecular assays that are essential to exploit all possibilities coming from our results.

In summary, we have found that inhibition of ADA increases the signal amplification of the A_1_ adenosinergic system as regards the direct negative inotropic effect in the euthyroid and, a fortiori, hyperthyroid guinea pig atrium. This outcome indicates that ADA inhibition, besides producing an increase in the interstitial adenosine level with a consequent stimulation of the A_1_ receptor, intensifies the A_1_ adenosinergic direct negative inotropic function in another way (for as much as the extra adenosine can evoke a stronger effect if using a more efficacious signaling). Thus, our results propose a new thyroid hormone-sensitive mechanism of action of ADA inhibition that may have practical significance in improving ischemic tolerance of the heart. Of course, this practical impact depends on whether this phenomenon affects other A_1_ receptor-mediated protective functions as well and whether it extends to the whole heart. It is especially interesting that this action of ADA inhibition is stronger in hyperthyroidism, a condition that places an extra burden on the heart with a simultaneous reduction of some A_1_ receptor functions. In addition, it has been concluded that the site of the efficiency-enhancing action of ADA inhibition is not located in the joint part of signaling pathways of A_1_ and M_2_ receptors. Furthermore, it has been found that ADA inhibition can produce a smaller rise in the interstitial adenosine concentration than nucleoside transport blockade can in both eu- and hyperthyroid atria.
